# Evidence for the presence of African swine fever virus in apparently healthy pigs in South-Kivu Province of the Democratic Republic of Congo

**DOI:** 10.1016/j.vetmic.2019.108521

**Published:** 2020-01

**Authors:** Bisimwa N. Patrick, Eunice M. Machuka, Dedan Githae, Gédéon Banswe, Joshua O. Amimo, Juliette R. Ongus, Charles Masembe, Richard P. Bishop, Lucilla Steinaa, Appolinaire Djikeng, Roger Pelle

**Affiliations:** a Pan African University Institute of Basic Sciences, Technology and Innovation, Department of Molecular Biology and Biotechnology, Nairobi, Kenya; b Biosciences Eastern and Central Africa-International Livestock Research Institute (BecA-ILRI) Hub, Nairobi, Kenya; c University of Nairobi, Department of Animal Production, Nairobi, Kenya; d Jomo Kenyatta University of Agriculture and Technology, Department of Medical Laboratory Sciences, Nairobi, Kenya; e Makerere University, Department of Zoology, Entomology and Fisheries Sciences, Kampala, Uganda; f Washington State University, Department of Veterinary Microbiology and Pathology, Pullman, WA, USA; g International Livestock Research Institute, Department of Vaccine Biosciences, Nairobi, Kenya; h Université Evangélique en Afrique, Department of Animal Science and Production, Bukavu, DR, Congo

**Keywords:** Diagnosis, African swine fever, Asymptomatic pigs, Transmission, South Kivu province

## Abstract

African swine fever (ASF) is the most important disease constraining smallholder pig production in the Democratic Republic of Congo, causing high mortality in domestic pigs with severe impacts on the livelihoods of local populations. This study was conducted with the aim of determining the prevalence of ASF and circulating virus genotypes in asymptomatic pigs raised on smallholder farms in the South Kivu province to understand the transmission dynamics of ASF and ultimately improving disease control. A cross-sectional survey was carried out in 5 districts where 267 pig blood were screened for both antibody and viral genome using indirect Enzyme Linked Immunosorbent Assay (ELISA) and polymerase chain reaction (PCR) respectively. Additionally, amplicons from PCR positive samples were sequenced by Sanger method for genetic analysis of ASF virus (ASFV) based on the C-terminal region of the B646L gene that encodes the major capsid protein p72 and the gene E183L encoding the p54 protein. The overall seroprevalence obtained based on antibody to p30 protein was 37 % and was significantly higher (P < 0.05) in adult (>1 year) animals (44.7 %) than in younger (<1 year) ones (33.5 %). Moreover, the seropositivity varied significantly (P < 0.05) according to the pig husbandry system practiced within the districts investigated with Uvira (55 %) and Mwenga (42.2 %) having the highest ASFV antibodies, while the lowest (10.5 %) were in Kalehe. Free-range pigs exhibited a higher level of seropositivity to ASFV antibody (68.9 %) than pigs kept in the pigsty housing system (21.6 %). However, no statistically significant differences (P > 0.05) were observed when sex of the animal and breed were factored. PCR detection of ASFV amplified a specific band of expected size (257 bp) in 61 out of 267 blood samples, confirming the presence of the viral DNA in 22.8 % of asymptomatic domestic pigs. Statistical analysis revealed that ASFV infection in domestic pigs varied significantly (p < 0.001) according to geographical location and breed, with the highest infection rate found in Walungu district (33.7 %) while the lowest was registered in Kalehe (15.8 %). Local pigs (27.2 %) were more infected than crosses (9.2 %).

Phylogenetic analyses based on partial sequences of the p72 and p54 genes revealed that all the ASFV detected belonged to genotype IX, which has previously been reported in other parts of DR Congo, and was clustered together with isolates from Kenya, Uganda and Republic of Congo. This study avails the first evidence of the presence of ASF virus in domestic pigs in the absence of outbreaks in South Kivu province, eastern DR Congo, indicating a need to raise awareness among pig farmers and veterinary authorities on the application of biosecurity measures and good husbandry practices to control the disease.

## Highlights

• The high level of antibody in adult animal kept in free-range system emphasis use of good husbandry practices.
• The presence of viral DNA in apparently healthy animals help in understanding the persistence of ASF.
• The differences in ecological conditions may play a key role in virus transmission.
• Identification of ASFV genotype IX confirms spread of the virus throughout the country.

## 1 Introduction

Disease is one of the most important constraint that affect the livestock production in several countries in Africa (Abubakar et al., 2003). Pigs are affected by a range of diseases and pests, some of which are zoonotic while others are transboundary such as African swine fever (ASF). ASF is a complex contagious viral disease affecting domestic pigs with considerable economic loss due to morbidity and death of animals with mortality approaching 100 % in naïve herds (Jori and Bastos, 2009). Furthermore, the risk of such losses may constrain some smallholders from investing in the potentially profitable pig farming sector. The disease is classified as a notifiable disease by the World Organization for Animal Health (OIE). ASF is caused by the ASF virus (ASFV), which possesses a large double-strand DNA genome and belongs to the genus of *Asfivirus*and family *Asfarviridae* (Penrith et al., 2005). The disease is endemic in Sub-Saharan Africa and has been reported in more than 26 countries with serious implication for food and nutritional security by limiting the availability of an important source of human dietary protein as well as high impact on the incomes of smallholder farmers when pigs are lost (Costard et al., 2009b). The potential spread of ASF and the increasing occurrence of inter-continental contamination transmission through contaminated pork products, exemplified by the recent pandemic caused by a genotype II virus originating in South East Africa and reaching the Black Sea region and subsequently Russia, Eastern Europe and China, affect the pig industry worldwide and also limit African farmers from exporting to international markets (Costard et al., 2009a). Currently, ASFV is the only known DNA arbovirus. The vectors for ASFV are soft ticks in the family Argasidae classified within the genu *Ornithodoros* spp. The virus was initially discovered to infect *Ornithodorus erraticus* ticks following introduction of ASFV genotype I from Angola to the Iberian Peninsula (Penrith and Vosloo, 2009; Penrith et al., 2004a) and subsequently confirmed in Africa in *Ornithodorus moubata* ticks present in warthog (*Phacochoerus aethiopicus* and *P. africanus*) burrows (Plowright et al., 1994). Susceptible animals are infected through either the sylvatic or the domestic cycles (Okoth et al., 2013). In the sylvatic cycle, the ASF virus circulates between African wild pigs particularly warthogs and bush pigs which are wildlife reservoirs and soft ticks of the *Ornithodoros* spp, without any apparent clinical sign of disease in these African wild pigs (Costard et al., 2009b). The domestic cycle occurs when the virus is transmitted directly from one domestic pig to another or from pig products to domestic pigs, without the involvement of sylvatic hosts or arthropod vectors (Costard et al., 2009b; Jori et al., 2013). In addition, several studies reported a direct transmission between infected bush pigs and domestic pigs, and between pigs-to-pigs in domestic cycle through contact (Penrith et al., 2004b; Costard et al., 2009a, b). No vaccine or chemotherapeutic treatment are currently available to treat clinically symptomatic animals, or to prevent the spread of the disease. Current efforts to develop vaccines are hampered by the high genetic diversity of ASFV and its capacity to modulate the phenotype of infected host macrophages, as well as the reported absence of neutralizing antibodies in infected pigs (Sánchez-Vizcaíno et al., 2013). Prevention and control are almost exclusively based on the strict application of biosecurity measures and stamping out infected swine herds through culling (Penrith et al., 2004b).

During ASFV epidemics, most of infected pigs die because of the disease. However, in some pigs recovered from ASF infection, the virus can still be detected in both tissue and blood of the surviving pigs (carriers), persists for long period and these clinically healthy carrier pigs can transmit ASFV to other naïve animals through direct contact resulting in acute onset of disease in new population (Eblé et al., 2019).

In central Africa region, DR Congo has the second largest pig population standing at about 1 million units (INS, 2015), distributed unequally in different provinces across the whole country including South Kivu. Although pig farming is considered an important livestock activity for many farmers in several rural areas of South Kivu, this region is still among the provinces with a low pig population compared to other regions of the country, because of the frequent occurrence of ASF outbreaks with high mortality. However, several pigs have been observed to survive after outbreaks and these may have acquired immunity to ASF due to prolonged and/or repeated exposures to low doses of the virus or challenge with viruses of low infectivity and virulence. These recovered animals could be reservoirs that may be capable of infecting naïve animals (Mulumba-Mfumu et al., 2015). Despite the lack of knowledge relating to the frequency of ASFV persistence in the endemic region, the evidence that recovered animals can transmit the virus to naïve populations for up to 6 months post-infection has been documented (Gallardo et al., 2015b; Costard et al., 2009b; Eblé et al., 2019) resulting in new outbreaks (Bech-Nielsen et al., 1995).

South Kivu province is an area in the eastern DR Congo where suspected outbreaks have been regularly reported with several pigs surviving throughout outbreaks. However, there is lack of knowledge regarding the frequency of ASFV in asymptomatic pigs and the genetic nature of ASF viruses circulating in the different parts of this region. This study attempts to address these gaps by determining the seroprevalence of ASF by screening pigs for ASFV antibodies as well as for the presence of the viral genomic DNA and analysing the genetic diversity of circulating ASFV strains in the South Kivu province. Our findings contribute to determine the frequency of viral infections and the potential for disease spread. Ultimately, this study will contribute to the development of appropriate management strategies for the ASF in the South Kivu region.

## 2 Materials and methods

### 2.1 Study areas

**Fig. 1:**
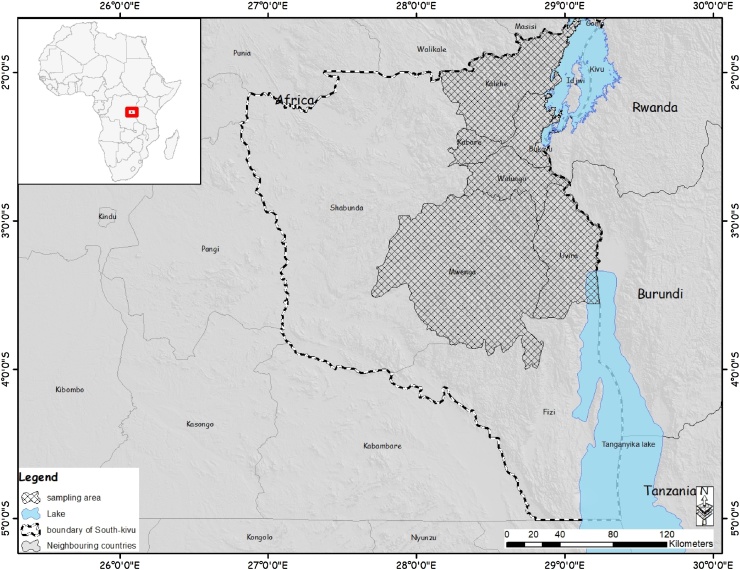
Map of South Kivu province showing sampling locations for African swine fever (Draw with Arc-GIS).

The study was conducted in five different districts in the South Kivu province located in the Eastern part of the Democratic Republic of Congo including Kabare, Kalehe, Mwenga, Uvira and Walungu (Fig. 1). This is a vast region with a surface of 66.814Km^2^, located between longitudes 26° 10′ 30″ and 29° 58′ east, and latitudes 00′ 58″ North and 4° 51′ 21″ South. There are 9 months of the rainy season (September up to May) and 3 months of the dry season from June to August, with an annual average rainfall of about 1300 mm. These districts were selected following consultation with the Ministry of Agriculture Livestock and Fishery because of the important pig keeping activity, reported outbreaks of ASFV and accessibility of these localities.

### 2.2 Study design, sample size determination and sample collection

A cross-sectional study was conducted on pig farms across the five districts in South Kivu province to estimate ASF seroprevalence during an eight months period from January to August 2016. The survey involved a total of 250 pig farmers, randomly selected with support from the Veterinary Services Department of Veterinary Services of the Ministry of Agriculture, Livestock and Fishery in collaboration with pig breeder organizations, who provided a list of all pig owners present in the region.

The target population comprised all pig keepers having experience of more than 2 years in pig farming and those that had experienced the outbreaks of the disease for the last two years.

The sample size for pigs that achieved 5 % level of precision at 95 % confidence level was determined using the formula: n = Z^2^ X P (1-P)/d^2^, where n = sample size, Z = 1.96 at 95 % confidence level, P = probable prevalence of ASFV antibody in pig herds estimated at 50 %, d = standard error (taken as 6 %) (Dohoo et al., 2009).

Animals on which blood sample was collected included domestic pigs (male and female) that have survived outbreaks and those with no clinical signs specific to ASFV. Additionally, all pigs older than 3 months to ovoid maternal antibodies, sow having a litter of more than 3 months old as well as non-pregnant sows. However, a pig was excluded for the study if less than 3 months old because of the technical difficulties of drawing blood samples, pregnant sow, sow having a litter of less than 3 months old as well as when the owner has not been located. Based on the formula and selection criteria above, a total of 267 pigs were successfully sampled with overall of 1–2 pigs considered par farm.

Plain tubes were used to collect approximately 3−5 ml of blood aseptically from the jugular vein from all pigs over three months of age, except when they were heavily pregnant or had recently farrowed, or the owner could not be located. After collection, all samples were transported unrefrigerated to the molecular biology laboratory at the Evangelical University in Africa, where blood samples were stored at room temperature for 30−60 min to clot followed by centrifugation at 3000 rpm for 10 min to recover the serum which was stored at -20 °C before being shipped to BecA-ILRI Hub in Nairobi, Kenya for serological analysis. Similarly, a total number of 267 whole blood samples were collected on Whatman FTA cards for this study. Dried FTA blood was stored at room temperature before being transported to BecA-ILRI Hub for molecular analysis.

### 2.3 Collection of epidemiological data

Interviews were conducted with pig owners in the 5 selected districts with the aid of a structured questionnaire designed to elicit information about the history of ASF occurrence on the farm, breed of pigs present on the farm, the biosecurity practices employed. Evidence for free-range husbandry, and the feeding systems used were also recorded. Farmers were also shown an *Ornithodoros* tick to elicit information on whether there was a perception that Argasid soft ticks were present on the farms selected for pig sampling.

### 2.4 Laboratory analysis

#### 2.4.1 Screening for antibodies

Immunoglobulin G antibodies against ASF virus was detected in serum samples by an indirect enzyme-linked immunosorbent assay (ELISA) using the SVANOVIR® ASFV-Ab kit, (ALGENEX Madrid, Spain) following the manufacturer’s instructions. Briefly, 100 μl of pre-diluted positive control serum and 100 μl of pre-diluted negative control serum diluted 1/100 in sample dilution buffer, was added to the 96-well microtiter plates (Maxisorb, Nunc, Denmark) coated with ASFV antigen. These samples were diluted as the controls and added in duplicates and then mixed as recommended followed by incubation at 37 °C for 1 h.

After washes with PBS-Tween-20 buffer, 100 μl of horseradish peroxidase conjugated anti-swine antibody was added in each well and plates were incubated at 37 °C for 1 h. The plate was washed and 100 μl of substrate solution were added to each well. After incubation for 10 min at room temperature, the reaction was stopped by adding 50 μl of the stop solution to each well. Optical density (OD) was then determined at 450 nm in the microplate reader (Biotek synergy HT, US),15 min after the addition of stop solution to prevent fluctuation in OD values. The percentage positivity (PP) values were determined and interpreted using the formula presented in the protocol supplied by the company. Samples with a PP ≤ 10 were considered negative, while samples with PP > 10 were considered to be positive. According to the manufacturer, the sensitivity and specificity of this kit are 100 % and 92.5 %, respectively, and can detect antibodies from day seven post-infection.

#### 2.4.2 Molecular analysis

##### 2.4.2.1 DNA extraction

DNA was extracted from FTA blood samples using QIAamp kit (INVISTA North America S.A.R.L. Corporation) following the manufacturer’s recommendations. Briefly, 6 mm of a sample disc was removed from the centre of a dried blood sample spot using Harris Uni-Core disposable punch and placed into a 1.5 ml microcentrifuge tube. A volume of 280 μl of tissue lysis buffer (ATL) and 20 μl of Proteinase K was added to the tube containing the disc followed with pulse vortexing for 30 s. The mixture was heated at 56 °C for 60 min with vortexing steps for 30 s every 10 min. afterwards, 300 μl of lysis buffer (AL) was added and vortexed followed by an incubation at 70 °C for 10 min with vortexing for 10 s every 3 min. The mixture was centrifuged at 20,000 x g for 30 s, the lysate was transferred to a mini Elute column and then centrifuged at 6000 x g for 1 min before discarding the flow through. Mini Elute columns were washed twice with 700 μl of wash buffer 1 (AW1) and wash buffer 2 (AW2) followed by centrifuging for 1 min at 6000 x g, and discarding the flow through; the second washing step followed using AW2 buffer 700 μl of 100 % ethanol was added to the min Elute column and centrifuge for 1 min at 6000 × g. The columns were left to stand for 10 min at room temperature after discarding the flow through, followed by centrifugation at 20,000 x g for 3 min. DNA was finally eluted into 1.5 ml Eppendorf tube by addition of 40 μl nuclease free water, incubated for 2 min at room temperature and centrifuged at 6000 x g. Eluted DNA was stored at −20 °C until use.

##### 2.4.2.2 Detection of African swine fever virus

A 257 bp region corresponding to the C-terminal region of the B646 l gene encoding the p*72* major capsid protein was amplified using the diagnostic primers PPA1 and PPA2 as previously reported (Aguero et al., 2003) to confirm the presence of the ASFV nucleic acid. The PCR were performed in a total volume of 25 μl containing 1.25 μM of each primer, OneTaq 2x MasterMix using a standard buffer (New England Biolabs, UK) which contains (1.8 mM MgCl_2_, 0.2 mM dNTPs, 25 units/ml OneTaq DNA polymerase, 20 mM Tris−HCl) and additional 2.5 mM of MgCl_2_ added. DNA was amplified at 95 °C for 10 min as initial denaturation followed by 40 cycles of denaturation at 95 °C for 15 s, annealing at 62 °C for 30 s and extension at 72 °C for 30 s. These cycles were followed by an extension step of 72 °C for 7 min. PCR products were analyzed by gel electrophoresis on a 1.8 % agarose gel stained with GelRed nucleic acid stain and visualized using UV light.

##### 2.4.2.3 Phylogenetic and sequence analysis

To characterize ASF virus, 11 out of the 61 positive samples were randomly selected, amplified and sequenced by Sanger method using epidemiological primers p72-U (5′-GGCACAAGTTCGGACATGT-3′) and p72-D (5′-GTACTGTAAC GCAGCACAG-3′) which amplify a 478 bp product (Bastos et al., 2003). DNA amplification was performed as follows: initial denaturation at 95 °C for 10 min followed by 40 cycles of denaturation at 95 °C for 30 s, primer annealing at 48 °C for 30 s, extension at 72 °C for 1 min followed by a final extension at 72 °C for 10 min as reported (Gallardo et al., 2011). Amplicons of the expected size were purified using QIAquick PCR purification Kit (Qiagen, Germany) following the manufacturer's recommendation and sent for sequencing (Macrogen Inc, Geumcheon-gu, South Korea). The resulting sequences were trimmed, assembled and quality checks were done using CLC main Work Bench version 7.8.1 software. Sequences were compared with sequences of ASFV reference strains in the GenBank database by BLAST (http://blast.ncbi.nlm.nih.gov/Blast.cgi). Multiple alignments were created by using the ClustalW ((http://www.ebi.ac.uk/Tools/msa/clustalo/) which included sequences obtained from the eastern DR Congo pig blood DNA samples and ASFV sequences available in GenBank. The p72 sequence alignment was subsequently used for phylogenetic analyses. A phylogenetic tree was constructed by the maximum likelihood method using the Kimura-2-parameter option generated using MEGA 7 (Kumar et al., 2016) with 1000 bootstrap replications. The phylogenetic tree was drawn to scale, with branch lengths equivalent to the evolutionary distances used to infer the relationship between the sequences. All the sequences generated from this study have been submited to the GenBank uner accession number MN609932-MN609942 (p72) and MN615716 - MN615721(p54).

### 2.5 Statistical analysis

We conducted descriptive statistical and univariate analysis using CDC Epi-info™ version 7 (Centers for Disease Control and Prevention, 2016). The odds of an ASF case based on serology or PCR results was then modeled as a function of the dichotomous risk factor measures, using conditional logistic regression models. The odds ratio and statistical significance between positive and negative pig farms were determined using Fisher’s exact test for discrete variables using the 95 % confidence level.

## 3 Results

### 3.1 Phenotypic characterization and production system of domestic pigs sampled in the field survey in South Kivu province, East of Democratic Republic of Congo

During this study, 250 pig farmers were interviewed, including 181 (72.4 %) were females with an age above 25 years. Most respondents, 153 (61.2 %), had primary school education level and most of the farms, 174 (69.6 %), had less than two years’ experience in pig faming and husbandry practice (Data not shown).

In total, 267 domestic pigs from 5 districts of the South Kivu province were sampled and tested for the presence of ASFV antibodies and ASFV DNA. Approximately 202 (75.6 %) of the animals were local breed and the rest were exotic (European breed); more than half of the pigs, 172 (64.4 %), were female animals and most of them, 181(67.8 %), were adult (above 1 year old). The highest number of pigs, 101 (39.3 %), were from the Walungu district and many of them (67.8 %) were kept in housing husbandry system. The major source of feeding was mainly the use of waste from house (90.7 %). However, ticks were observed on only 32.6 % of the pigs and most of the animals (79.4 %) were not from recent outbreak. Moreover, the majority of pigs examined (96.3 %) have been in contact with a neighbor farm (Table 1).

**Table 1:** Characteristics of clinically healthy pig populations screened in each district.

Variables	Category	Kabare n = 64(%)	Kalehe n = 19(%)	Mwenga n = 63(%)	Uvira n = 20(%)	Walungu n = 101(%)	Total n = 391(%)
Age	Adult (>1 year)	13(20.3)	3(15.8)	33(52.4)	9(45)	27(26.7)	85(31.8)
	Young(<1 year)	51(79.7)	16(84.2)	30(47.6)	11(55)	74(733)	182(68.2)
Sex	F	45(70.3)	15(79)	41(65)	12(60)	59(58.4)	172(64.4)
	M	19(29.7)	4(21)	22(35)	8(40)	42(41.6)	95(35.6)
Husbandry system	Free-rage	10(15.6)	1(5.3)	43(68.2)	19(95)	13(12.9)	86(32.2)
	Housed	54(84.4)	18(94.3)	20(31.7)	1(5)	83(82.2)	181(67.8)
Breed	Exotic	19(29.7)	15(78.9)	13(20.6)	6(30)	18(17.8)	86(32.2)
	Local	45(70.3)	4(21)	50(79.4)	14(70)	89(88.1)	202(75.6)
Origin food	Field	37(57.8)	7(36.8)	25(39.7)	12(60)	60(59.4)	141(52.8)
	Market	8(12.5)	2(10.5)	7(11.1)	0(0)	8(7.9)	25(9.3)
	Waste from house	19(29.7)	10(52.6)	31(49.2)	8(40)	33(32.7)	242(90.7)
Presence of ticks	No	49(76.6)	16(84.2)	22(34.9)	12(60)	81(80.2)	180(67.4)
	Yes	15(23.4)	3(15.8)	41(65)	8(40)	20(19.8)	87(32.6)
Pigs kept near abattoir	No	42(65.6)	9(47.3)	54(85.7)	20(100)	101(100)	226(84.6)
	yes	22(34.3)	10(52.6)	9(14.3)	0(0)	0(0)	41(15.6)
Recent outbreak	No	59(92.1)	18(94.7)	50(79.4)	6(30)	79(78.2)	212(79.4)
	Yes	5(7.8)	1(5.3)	13(20.6)	14(70)	22(21.8)	55(20.6)
New animal intr. In farm	No	16(25)	5(26.3)	17(27)	8(40)	52(51.5)	98(36.7)
	Yes	48(75)	14(73.7)	46(73)	12(60)	49(48.5)	169(63.3)
Move pig to neighbour farm	No	3(4.7)	0(0)	2(3.2)	1(5)	4(4)	10(3.7)
	Yes	61(95.3)	19(100)	61(96.8)	19(95)	97(96)	257(96.3)

### 3.2 Seropositivity for African swine fever virus

Of the 267 pigs tested for ASF virus antibodies, 99 were positive using the indirect ELISA resulting in an overall seroprevalence of 37 % (OR = 4.7; 95 % CI: 2.67–8.28). As shown in Table 2, the sero-positivity varied significantly according to age and husbandry system (p < 0.05). The sampling number varied according to districts and the sero-prevalence of ASFV antibody was different between districts. The higher seroprevalence (45.8 %) was found in the adults pigs (≥ 1 year), when compared to the younger (< 1 year) pigs (32.9 %). The seroprevalence was notably higher in pigs kept in free-range systems (68.9 %) as compared to those in housing systems (21.6 %). The highest seroprevalence (55 %) was observed in Uvira district (55 %; OR = 10.3; 95 %CI: 0.01-0.53), followed by Mwenga (46 %; OR = 7.2; 95 %CI: 0.02-0.64), Walungu (37.6 %; OR = 5.2; 95 %CI: 0.04-0.89), and the lowest in Kabare (34.3 %; OR = 4.4, 95 %CI: 0.04–1.06).

**Table 2:** African swine fever seroprevalence in asymptomatic pigs in South Kivu province (DRC) with statistical analysis using Chi-square test.

Variables	Characteristics	Sero-positive animals based on X^2^ test				OR	95 % CI	P-value
		Positive	%	Negative	%			
Sex	Male	31	32.6	64	67.4	1	–	–
	female	68	39.5	104	60.5	1.35	0.43-1.25	0.325
Age	Adult	38	44.7	47	54.3	1	–	–
	Young adult	61	33.5	121	66.5	0.58	1.01-2.91	0.0302
Breed	Local	74	36.8	127	63.2	1	–	–
	Exotic	25	37.8	41	62.2	1.04	0.53-1.68	0.993
Husbandry system	Free-range	60	68.9	27	31.1	1	–	–
	Housed	39	21.6	141	78.4	8.03	4.51-14-29	0.0001
Districts	Kalehe	2	10.5	17	89.5	1	–	–
	Kabare	22	34.3	42	65.7	4.45	0.04-1.06	0.086
	Mwenga	26	41.2	37	58.8	5.97	0.03-0.78	0.028
	Uvira	11	55	9	45	10.38	0.01-0.53	0.008
	Walungu	38	37.6	63	62.4	5.27	0.04-0.89	0.042

OR: Odds ratio; CI: Confidence Interval; X^2^: Chi–Square.

However, no statistically significant differences were observed between sex and breed (P > 0.05).

### 3.3 Polymerase chain reaction positivity for African swine fever virus

A total of 267 blood samples collected from clinically healthy pigs were screened for the presence of the ASF viral DNA using conventional PCR with diagnostic primers PPA1/PPA2. A total of 61 blood samples out of 267 analyzed were ASFV positive, giving a prevalence of 22.8 % (95 % CI, 2.7–9.5). The statistical analysis revealed that ASFV infection in domestic pigs varied significantly according to breed (P = 0.003) and geographical location (P < 0.0001). The prevalence was higher in local breed pig than in crosses with commercial breeds (improved) pigs (27.2 %; OR = 3.67; 95 %CI: 0.11-0.66). Based on districts, the prevalence of ASFVwas highest in Walungu district (33.7 %; OR = 15.7; 95 %CI: 0.01-0.27), followed by Mwenga district (28.6 %; OR = 12.4; 95 %CI: 0.01 - 0.36), Uvira (20 %; OR = 7.7; 95 %CI: 0.02–0.76) while the lowest was Kalehe (15.8 %; OR = 5.8; 95 %CI: 0.02–1.11). In addition, our results revealed that female pigs were more likely to be ASFV positive when compared to males (OR = 1.07; 95 % CI: 0.56–1.86); whilst the young adults (OR = 1.57; 95 % IC: 0.33–1.21) and pigs kept in housed husbandry system (OR = 1.03; 95 % IC: 0.56–1.90) were more likely to be infected by ASFV than adult and pigs keeping in free-range system (Table 3). Out of the 99 animals that were anti-ASFV antibody positive (seropositive), 59 (22 %) were PCR positive. Additionally, 2 pigs which were seronegative were found to be positive by PCR assay (Table 4). A significant association (p < 0.0001) was found between antibody-positive and PCR-positive for which most of the animals seropositive were also PCR positive.

**Table 3:** ASFV infection using PCR assay in domestic pigs according to sex, age, breed, husbandry system and geographical location in South Kivu province, eastern DRCongo.

Variables	Characteristics	No. pigs tested	Positives		Negatives		OR	95 % CI	P-value
			n	%	n	%			
Sex	Male	95	22	23.2	73	76.8	1	0.56 - 1.86	0.928
	Female	172	39	22.7	133	77.3	1.07		
Age	Adult (>12months)	85	15	17.6	70	82.4	1	0.33-1.21	0.167
	Young adult (<12months)	182	46	25.3	136	74.7	1.57		
Breed	Crosses	65	6	9.2	59	90.8	1	0.11- 0.66	0.003
	Local	202	55	27.2	147	72.8	3.67		
Husbandry system	Free-rage	87	20	23.0	66	75.9	1	0.56 – 1.90	0.913
	Housed	180	41	22.8	140	77.8	1.03		
Districts	Kabare	64	2	3.1	62	96.9	1	–	–
	Kalehe	19	3	15.8	16	84.2	5.8	0.02- 1.11	0.07
	Mwenga	63	18	28.6	45	71.4	12.4	0.01- 0.36	0.0002
	Uvira	20	4	20.0	16	80.0	7.7	0.02- 0.76	0.04
	Walungu	101	34	33.7	67	66.3	15.7	0.01 – 0.27	<0.0001

OR: Odds ratio; CI: Confidence Interval; X^2^: Chi –square; n: number of sample tested.

**Table 4:** Association between antibody-positive and PCR-positive domestic pigs.

		PCR		
		+	–	Total
ELISA	+	59 (22)	40 (15)	99 (37)
	–	2 (1.2)	166 (62.2)	168 (63)
	Total	61 (22.8)	206 (77.1)	267 (100)

Moreover, the infection status varied depending on the geographical locations where by most of samples tested seropositive in all districts were also PCR–positive except in districts such as Kabare and Uvira where 2 out of 22 seropositives and 4/11 samples tested ASFV-antibody positive were PCR-positive respectively.

### 3.4 Nucleotide sequence and phylogenetic analysis

**Fig. 2:**
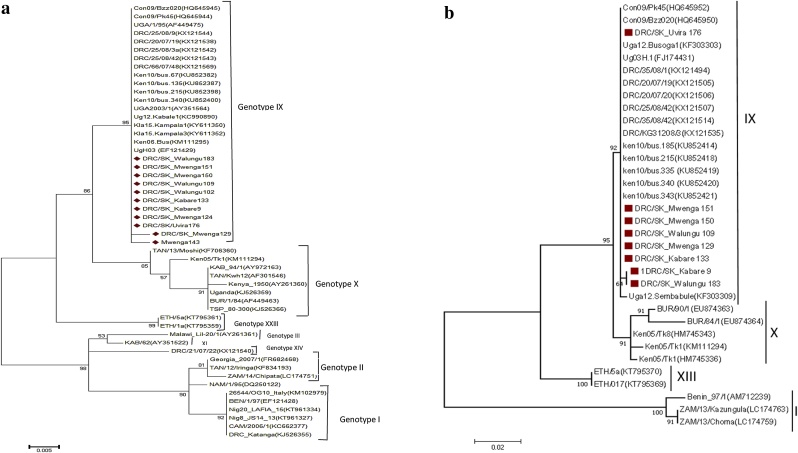
a Phylogenetic relationship between ASFV strains analyzed in this study and previously identified ASFV genotypes depicted as Maximum likelihood tree. The numbers at nodes represent the percentage of 1000 bootstrap replicates. The scale bar indicates the number of nucleotide substitutions per site. The p72 sequences isolated in this study are marked with red point. Isolated are named in the following manner: Country, isolate name, accession number. Figure 2b Phylogenetic tree based on the full length E183 l gene (p54). Indicates the 20 sequences analyzed in this study are indicated by red dot and are clustered within genotype IX. The evolutionary history was inferred using the Minimum Evolution method based on the Kimura-2 parameter model. The percentage of replicate trees in which the associated taxa clustered together in the bootstrap test (1000 replicates) are shown next to the branches. The tree is drawn to scale with branch lengths in the number of substitutions per site. (For interpretation of the references to colour in this figure legend, the reader is referred to the web version of this article).

Out of the 61 PCR positive samples sent for sequencing, only 11 amplicons have generated successfully good quality sequence reads that were able to be analysed by the software. Results of the comparative analysis of the p72 nucleotide sequences of the ASFV detected in apparently healthy domestic pigs from the different districts of the South Kivu province revealed that they were 100 % identical to one another at the nucleotide level. Blast results of the B646 l (p72) South Kivu ASF virus DNA sequences from Genbank showed 99 % nucleotide similarity with p72 sequences of Congolese isolates such DRC/25/08/9, DRC/25/08/3a, DRC/25/08/42 and DRC/20/07/19. Additionally, the phylogenetic analysis based on both p72 and p54 revealed that the South Kivu ASF viruses belonged to genotypes IX and were 98 % identical with some ASF viruses which have been reported in previous studies in different parts of Uganda, Kenya and Republic of Congo and clustered together (Fig. 2a and b).

## 4 Discussion

During this investigation of ASFV infected, but apparently asymptomatic pigs, 267 serum samples collected from apparently healthy pigs were examined for the presence of African swine fever virus antibodies and ASFV DNA. The farmer survey data revealed that (61.2 %) of pig keepers interviewed had achieved primary school education level and most of them (69.6 %) had less than 2 year experience in pig faming and husbandry practice for less than 2 years. The limited experience and limited education might had a negative impact on pig management and therefore increase the likelihood of disease occurrence because most of the farmers do not apply proper biosecurity measures or husbandry practices husbandry practices (Nantima et al., 2015). These results are in concordance with recent studies conducted in Uganda (Dione et al., 2015; Nantima et al., 2015) which found that insufficient knowledge of husbandry practices and pig management were among the priority constraints faced by pig farmers in Uganda. From this study, an ASFV seroprevalence of 37 % (99/267) was observed which is relatively high compared to the 13.3 % reported in Taraba state, northeastern of Nigeria (Abwage et al., 2015), 0 % in South-west and central Kenya (Okoth et al., 2013; Abworo et al., 2017), 2.1%–12.5% in southern Malawi and 0 % in central Uganda (Allaway et al., 1995; Muhangi et al., 2015). The dissimilarities of the regional findings could be probably explained by the type of husbandry system used as well as the circulating regional ASFV strains. Additionally, the higher seropositivity could be possibly due by the fact that when time progresses, the pigs can recover from the carrier state and remain serologically positive only (Eblé et al., 2019).

However, the seroprevalence reported in our study is lower than those reported in previous studies in Uganda and other countries (Atuhaire et al., 2013; Muwonge et al., 2012). It is worth noting that the high prevalence reported in the Ugandan (Allaway et al., 1995) has been disputed by other scientists who have worked in Uganda. Given the disparity in apparent ASFV genotype prevalence between eastern DRC and West Kenya and Uganda, it would be interesting to determine the complete genomes of the isolates from eastern DRC for comparative analysis and also look in depth at the genetics of the local pigs. Risk factors that are strongly associated with the seroprevalence were the age of the animal (old pigs (45.8 %) were more susceptible), husbandry system (pigs kept in the free-ranging system (60/87, 68.9 %) exhibited a higher prevalence of infection as well as geographical location. The high number of sero-positive in adult pigs (45.8 %) compared to younger pigs (32.9 %) is most likely caused by the longer lifespan and therefore increased risk of exposure to repeated ASFV infections leading to the persistence of antibodies and suggesting the circulation of a low virulence ASFV genotype strains, in contrast to western Kenya and eastern Uganda, where genotype IX viruses are highly virulent. The lack of significant association between breed (i.e. local vs exotic) and sero-positivity from this study is in line with findings of previous studies, indicating that the virus apparently does not exhibit any breed preference (Penrith and Vosloo, 2009). The free- range system is common in certain areas where farmers lack financial resources capability to construct housing or purchase feeds especially during dry season when there no crop farming activity. This situation results in pigs scavenging for food exposing them to the risk of disease transmission by increasing the likelihood of pig into contact with an infected animal, and biting from an infected tick (Nantima et al., 2015; Dohoo et al., 2009).

The highest seroprevalence was reported in Walungu district followed by Mwenga. This may be due by the fact that Walungu samples were collected one month after suspected ASF outbreaks in the neighboring Mwenga district that led to high levels of pig movement between the two regions and may have contributed to the spread of disease from one area to another. Furthermore, the high seroprevalence obtained in domestic pigs is supported by previous studies revealing that pigs that survive natural infection after an outbreak develop antibodies against ASFV from 7 to 10 days after infection and are detectable after several months (Gallardo et al., 2015a; De Carvalho et al., 2012; Guinat et al., 2016; Sánchez-Vizcaíno et al., 2012). The high level of ASFV specific antibodies detected in this study (37 %) suggests that the disease might be endemic in this area but there is no formal reporting system in these areas. There is currently no documented evidence or that the circulating viruses are less virulent than those in other areas where genotype IX occurs. However, in areas where low virulence viruses genotypes are circulating, animals tend to have high ASF endemic status which leads to relatively high seroprevalence levels supporting this hypothesis (Carlos, 2009).

The conventional PCR results reported an overall ASFV prevalence of 22.8 % in the asymptomatic domestic pigs. This prevalence is higher than that reported in other studies conducted in Uganda and Western Kenya (Atuhaire et al., 2016; Etter et al., 2011; Gallardo et al., 2011; Okoth et al., 2013). This results was corroborated by the findings of Oluwole and Omitogun (2014) where ASFV genome was detected in Nigerian indigenous pigs infected with ASFV without showing any clinical symptoms. However, this is in contrast with another study conducted in Uganda where no viral DNA was detected despite the high apparent disease incidence (Muhangi et al., 2015).

The infection rate was found to be higher in indigenous breeds compared to cross bred animals; since this was a cross-sectional study, the pigs could have been chronic carriers that were sub-clinically infected or perhaps less likelyrecently infected with the virus but not showing the clinical signs. This observation is similar to the work carried out in Nigeria (Fasina et al., 2012) where ASFV has been detected in Nigerian indigenous pigs showing no symptom or clinical signs, although in this case, ASFV genotype I was involved and not genotype IX. Moreover, previous investigations demonstrated the ability of the virus to persist in virus tolerant African wild pigs while killing domestic hoste. For example a, case where a particularvirus was found in warthog and bush pigs but was absent in domestic pigs (Palgrave et al., 2011). Similar to our results, other studies have observed the tolerance of Nigerian indigenous pigs to ASFV for long periods without any clinical symptom or death (Adeoye and Adebambo, 2010). This is attributed to the ability of the host to sequester ASFV within macrophages in tissues other than the blood, where they are less exposed to host T cell responses (Adeoye and Adebambo, 2010).

The high ASFV infection rate observed in Walungu territory may be explained by the fact that a high number of pigs was sampled in this region compared to other territories, resulting in a more reliable prevalenceestimate. However, it is worth noting thata hemorrhagic disease outbreak suspected to be ASFV was reported in Mwenga territory which is neighbor to Walungu one month after sample collection with frequent movement of pigs observed between the two regions. The virus could quickly move from Mwenga to Walungu territory transported by free range pigs.This hypothesis is also supported by the fact that recovered that survive an outbreak retain virus for several months,withthe virus surviving in serum, blood, and other tissues, particularly the spleen. Such pigs can remain infective for several months (Gallardo et al., 2015b; Davies et al., 2015; Guinat et al., 2016).

Phylogenetic analysis of the ASFV strains obtained from this study based on the partial p72 gene clustered the ASF viruses from South Kivu province into genotypes IX. This ASFV genotype IX is known to be endemic to East and Central Africa, having been reported previously in Republic of Congo (Brazzaville), Uganda, and Kenya where it is involved in both sylvatic and domestic cycles (Lubisi et al., 2005; Gallardo et al., 2011). To our knowledge, this is the first molecular investigation of circulating ASF viruses in the South Kivu province; genotype IX was identified as the cause of ASFV outbreaks in Western, Central and Northern parts of DR Congo (Mulumba–Mfumu et al., 2017) and in Uganda as well (Gallardo et al., 2011). A similar genotype was identified in neighboring Republic of Congo where it is involved in both sylvatic and domestic cycles. Our data support previous studies which emphasize the role of neighboring countries in the epidemiology of the African swine fever (Atuhaire et al., 2013; Gallardo et al., 2011; Lubisi et al., 2005; Bastos et al., 2003). Despite the high prevalence of ASF in pigs detected in this study, it is thought that this virus may be circulating in porcine herds without clinical manifestation. This is amazing while genotype IX is generally known to be highly virulent (Gallardo et al., 2009). The fact that all the ASFV isolates from these findings are grouped into genotype IX based on p54 genotyping, it is suggested that the source of the virus may be from within the country or possibly from the eastern African region where this genotype is common.

## 5 Conclusion

Our findings constitute the first evidence demonstrating the presence of ASFV in apparently healthy domestic pigs from the South Kivu province of DR Congo with a relatively high prevalence of viruses within p72 genotype IX contrasting with previous studies in Uganda and Western Kenya. The prevalence within the Eastern DR Congo region was significantly different according to the age of pigs, husbandry system and the geographical location. Our data will ultimately be useful in contributing to an improved ASF control strategy in the region.

Screening of more samples will be required combined with genotyping at additional polymorphic loci to fully characterize ASFV in South Kivu province and it’s relatedness to other viruses within the region database.

## Ethics approval and consent to participate

A consent form which described the aim of the study was signed by farmers willing to participate in the study after translation into local languages. Ethical approval for the study reported here and the permission for the collection of samples was provided by the Interdisciplinary Centre for Ethical Research (CIRE) established by the Evangelical University in Africa, Bukavu, DR Congo, with reference (UEA/SGAC/KM 132/2016).

## Availability of data and material

The datasets used and/or analyzed during the current study are available from the corresponding author on reasonable request.

## Funding

This work was funded by the Biosciences Eastern and Central Africa - International Livestock Research Institute (BecA-ILRI) Hub, through Africa Biosciences Challenge Fund (ABCF).

## Declaration of Competing Interest

The authors declare no competing interests.
